# Data processing to support explication about effect of mineral constituents on temperature-dependent structural characterization of carbon fractions in sewage sludge-derived biochar

**DOI:** 10.1016/j.dib.2017.12.010

**Published:** 2017-12-13

**Authors:** Mi Li, Yuanyuan Tang, Nana Ren, Zuotai Zhang, Yiming Cao

**Affiliations:** a School of Environmental Science and Engineering, Southern University of Science and Technology, 1088 Xueyuan Blvd, Nanshan District, Shenzhen 518055, China; b Laboratory of Advanced Chemical Engineering, Dalian Institute of Chemical Physics (DICP), Chinese of Academy Sciences, No. 457 Zhongshan Road, Dalian, China; c Key Laboratory of Municipal Solid Waste Recycling Technology and Management of Shenzhen City, Shenzhen 518055, China

## Abstract

This dataset is the supplementary data for the summited research article Li et al., 2017 [1] and provides detailed data profiles to support the explication about mineral constituents’ effect on temperature-dependent structural characterization of carbon fractions in sewage sludge-derived biochar. The elemental compositions of major inorganics in the sewage sludge were detected by X-ray fluorescence spectrometry (XRF, S2-Ranger, Bruker).The images from scanning electron microscope (SEM) were compared between unwashed and acid-washed samples, and revealed the effect of acid washing on the surface morphology and porosity of sewage sludge and the biochar. Peak deconvolution was conducted for the (002) peak of X-ray diffraction (XRD) patterns from the acid-washed samples, which provided information on structural parameters of the carbon stacking structure and the temperature-dependent structure evolution of sewage sludge biochar. Peak deconvolution was also carried out for Raman data of the samples with/without consideration of mineral constituents (aluminosilicates). Results of Raman peak deconvolution showed structure ordering evolution with pyrolysis temperature and evidenced the contribution of mineral constituents to the Raman signals.

**Specifications Table**

**Table:** 

Subject area	chemistry
More specific subject area	structure of carbon
Type of data	Table, image, figure
How data was acquired	XRF: S2-Ranger, Bruker
	SEM: Zeiss Merlin operating at 3.0 kV
	XRD: Rigaku Smartlab powder X-ray diffractometer with the characteristic Cu Kα radiation and operating conditions of 35 kV and 40 mA
	Raman: Jobin Yvon LabRAM HR Evolution spectrometer with a laser excitation wavelength of 532 nm
Data format	Raw, analyzed
Experimental factors	– biochar was prepared from sewage sludge over a pyrolysis temperature range of 200–700 °C with a heating rate of 5 °C min^-1^ – raw sewage sludge and the obtained sewage sludge biochar were demineralized by washing with a mixture of 1 M HCl and 10% HF
Experimental features	Characterization data of unwashed and acid-washed samples from SEM, XRD and Raman are provided, together with the peak deconvolution of peaks from XRD patterns and Raman spectra
Data source location	Southern University of Science and Technology, Shenzhen, China
Data accessibility	Data are accessible with the article
Related research article	The associated research article related to this dataset is [1]

**Value of the Data**

• The elemental compositions of major inorganics in the sewage sludge were detected by XRF, showing Al, Si, Fe, and P as major inorganic elements.
• SEM images of unwashed and acid-washed samples show the effect of acid washing on surface morphology, and further help to reveal the effect of mineral constituents on physicochemical properties of the sludge-derived biochar.
• Peak deconvolution of XRD patterns has provided crystalline parameters for the carbon stacking structure, including the interlayer spacing of the crystallites, the crystallite size, and the structural orderliness of the carbon crystallite.
• Peak deconvolution of Raman spectra can demonstrate the structural ordering of the carbon fractions, further explicated the contribution of mineral constituents with consideration of Raman peaks from aluminosilicates.

## 1 Data

Table S1 shows the elemental compositions of major inorganics in the sewage sludge. Figure S1 demonstrates the SEM images of unwashed and acid-washed samples. Figure S2 shows peak deconvolution for the (002) peak of X-ray diffraction (XRD) patterns from acid-washed samples, while the corresponding structural parameters are summarized in Table S2. Figures S3-S6 illustrate peak deconvolution for the first-order Raman spectra of unwashed and acid-washed samples with or without consideration of aluminosilicates, and the corresponding parameters are given in Tables S3-S6.

## 2 Experimental design, materials and methods

Sewage sludge was collected from a municipal wastewater treatment plant at Shenzhen (China). The pyrolysis process for biochar production was carried out in a horizontal tube furnace over a pyrolysis temperature range of 200–700 °C at a heating rate of 5 °C min^-1^. The raw sewage sludge (SS) and the obtained sewage sludge biochar was demineralized by washing with a mixture of 1 M HCl and 10% HF, as described elsewhere [2], [3]. Detailed information on sample preparation can be found in [1].

The elemental compositions of major inorganics in the sewage sludge were detected by X-ray fluorescence spectrometry (XRF, S2-Ranger, Bruker). Surface morphology of the samples was observed by a SEM operating at 3.0 kV (Zeiss Merlin). The XRD pattern was recorded from 10° to 90° (2θ scale) at a scanning rate of 0.02° s^-1^ using a Rigaku Smartlab powder diffractometer with the characteristic Cu Kα radiation and operating conditions of 35 kV and 40 mA. The Raman experiments were conducted with a Jobin Yvon LabRAM HR Evolution spectrometer with a laser excitation wavelength of 532 nm from 800 to 2000 cm^−1^ with resolutions of 1–2 cm^-1^. Peak deconvolution for XRD and Raman data was carried out with software Fityk [4].

## References

[1] M. Li, Y.Y. Tang, N.N. Ren, Z.T. Zhang, Y.M. Cao Effect of Mineral Constituents on Temperature-Dependent Structural Characterization of Carbon Fractions in Sewage Sludge-Derived Biochar *J. Clean Prod.* 172, 2018, 3342-3350. 10.1016/j.dib.2017.12.010 PMC5988038 29876483

[2] Liu X.Q., Ding H.S., Wang Y.Y., Liu W.J., Jiang H. (2016). Pyrolytic temperature dependent and ash catalyzed formation of sludge char with ultra-high adsorption to 1-naphthol. Environ. Sci. Technol..

[3] Sun K., Kang M.J., Zhang Z.Y., Jin J., Wang Z.Y., Pan Z.Z., Xu D.Y., Wu F.C., Xing B.S. (2013). Impact of deashing treatment on biochar structural properties and potential sorption mechanisms of phenanthrene. Environ. Sci. Technol..

[4] Wojdyr M. (2010). Fityk: a general-purpose peak fitting program. J. Appl. Cryst..

