# Supplementary material for: Data processing to support explication about effect of mineral constituents on temperature-dependent structural characterization of carbon fractions in sewage sludge-derived biochar
Source: Data Brief. 2017 Dec 13;17:1304–6. doi: 10.1016/j.dib.2017.12.010 (PMC5988038; doi:10.1016/j.dib.2017.12.010)
Supplement: Supplementary file 1 — Supplementary material [file mmc1.docx]

Conflict of Interest

All the authors declares no conflict of Interest.
